# Ultrasonography‐driven combination antibiotic therapy with tigecycline significantly increases survival among patients with neutropenic enterocolitis following cytarabine‐containing chemotherapy for the remission induction of acute myeloid leukemia

**DOI:** 10.1002/cam4.1063

**Published:** 2017-05-26

**Authors:** Novella Pugliese, Paola Salvatore, Dora Vita Iula, Maria Rosaria Catania, Federico Chiurazzi, Roberta Della Pepa, Claudio Cerchione, Marta Raimondo, Claudia Giordano, Luigia Simeone, Simona Caruso, Fabrizio Pane, Marco Picardi

**Affiliations:** ^1^ Departments of Clinical Medicine and Surgery Federico II University Medical School Naples Italy; ^2^ Molecular Medicine and Medical Biotechnology Federico II University Medical School Naples Italy; ^3^ Advanced Biomedical Sciences Federico II University Medical School Naples Italy

**Keywords:** Acute myeloid leukemia, cytarabine, neutropenic enterocolitis, tigecycline

## Abstract

Neutropenic enterocolitis (NEC) is an abdominal infection reported primarily in patients with acute myeloid leukemia (AML) following chemotherapy, especially cytarabine, a notable efficacious cytotoxic agent for AML remission. Specific data regarding the impact of different cytarabine schedules and/or antibacterial regimens for NEC are sparse. The aim of the study was to identify the predictors of outcome within 30 days of NEC onset. NEC episodes were retrospectively pinpointed among 440 patients with newly diagnosed AML hospitalized in our Institution, over a 10‐year period, for receiving chemotherapy protocols with 100–6000 mg/m^2^ daily of cytarabine. Two subgroups, survivors versus nonsurvivors, were compared by using logistic regression analysis. NEC was documented in 100 of 420 (23.8%) analyzed patients: 42.5% had received high‐dose cytarabine, whereas 19% and 15% intermediate‐dose and standard‐dose cytarabine, respectively (*P* < 0.001). The 30‐day NEC attributable mortality rate was 23%. In univariate analysis, antileukemic protocols containing robust dosages of cytarabine were significantly associated with high mortality (*P* < 0.001); whereas, standard‐dose cytarabine and prompt initiation (at the ultrasonographic appearance of intestinal mural thickening) of NEC therapy with antibiotic combinations including tigecycline were significantly associated with low mortality. In multivariate analysis, high‐dose cytarabine‐containing chemotherapy was the independent predictor of poor outcome (odds ratio [OR]: 0.109; 95% confidence interval [CI]: 0.032–0.364; *P* < 0.001), whereas ultrasonography‐driven NEC therapy with antibiotic regimens including tigecycline was associated with a favorable outcome (OR: 13.161; 95% CI: 1.587–109.17; *P* = 0.017). Chemotherapy schedules with robust dosages of cytarabine for AML remission are associated with a high rate of NEC incidence and attributable. Vigorous antibacterial therapy, triggered off pathologic ultrasonographic findings, with drug combinations which have broad antimicrobial coverage and good gut penetration, specifically those also including tigecycline, may be effective in improving 30‐day survival rate after NEC onset.

## Introduction

Neutropenic enterocolitis (NEC) is a recognized infectious complication following cytotoxic agents: reported incidence rates range from 0.8% to 26% 1, 2. NEC is common in patients treated for acute myeloid leukemia (AML), especially those receiving intensive chemotherapy protocols with cytarabine 1, 2, 3. The diagnosis may be delayed due to the presence of clinical features such as neutropenic fever, abdominal pain, and/or diarrhea which are not specific and may suggest other abdominal diseases 1, 2. The majority of patients with NEC receive a definitive antiinfectious therapy in the absence of isolates to guide treatment 1, 2, 3. In fact, these patients fall within the scope of guideline recommendations concerning empirical treatment 1, 2. NEC therapy usually consists in the administration of *β*‐lactam agents, aminoglycosides, glycopeptides, and/or antifungal agents, according to the febrile neutropenia‐driven approach 1, 2, 4. However, a review of the literature indicates a considerable variability regarding the successful therapy of NEC: reported mortality rates range from 30% to 60% 2. Most deaths are due to necrotizing perforations, uncontrolled bleeding, abdominal abscess, and/or overwhelming sepsis. Small‐scale studies have revealed infiltrates of *Enterobacteriaceae* spp., *Enterococcus* spp., and other gram‐positive cocci and, rarely, *Pseudomonas aeruginosa* and *Candida* spp. in intestinal walls and peritoneal fluid at necropsy examinations 5, 6. Effective management of NEC is thus a considerable challenge for clinicians.

New imaging techniques and antibacterial drugs are now available for the management of infectious episodes in immunocompromised patients. Modern imaging methods are able to accurately define ileum and colon wall layers 6: gray‐scale ultrasonography (US) – a low‐cost and safe technique – can be easily performed and repeated at the bedside in severely ill patients 4, 7. US has proven useful in identifying intestinal mural thickening, the pathognomonic sign for NEC diagnosis according to the most widely accepted criteria 4, 8. Thickened intestinal wall reflects the pathology of the disease: destruction of normal mucosal and submucosal architecture of the lower intestinal tract with varying degrees of edema, ecchymosis, and erosion, with invasion of local tissue by intestinal microbes and subsequent peritoneal inflammation 1, 2. Tigecycline is the first agent in a new class of antibiotics belonging to the glycylcycline group, whose structure confers a broad antimicrobial spectrum with reported efficacy against a large number of gram‐positive, gram‐negative, anaerobic, and atypical pathogens, except *Pseudomonas aeruginosa* and *Proteus* spp 9. In a recent multicenter and randomized trial, Bucaneve et al. reported the adequacy of a front‐line tigecycline‐including regimen in a large group of high‐risk patients with neutropenic fever following treatment of acute leukemia 10. Importantly, the safety profile of tigecycline when investigated in patients receiving chemotherapies showed minimal organ toxicity and lack of dosage adjustment for hepatic or renal impairment 9, 10.

The aim of this study was to identify the predictors of outcome within 30 days of NEC onset. We provided an update on therapeutic interventions of NEC by performing a high‐quality evaluation with greater in‐depth analysis of several aspects of such complication. As a part of our Institutional guidelines for postchemotherapy supportive care 11, 12, 13, 14, high‐risk patients with neutropenic fever underwent strict diagnostic work‐up with ultrasonographic and microbiological assessment. NEC episodes were identified among a cohort of patients with AML undergoing chemotherapy protocols containing cytarabine in different schedules, then predictors of mortality and determinants of favorable outcome at 30 days after NEC onset were considered. Particular attention was focused on the impact of antibacterial regimens used in the different (empirical, US‐driven, and/or microbiology‐driven) phases of NEC treatment.

## Materials and Methods

### Study design and patients

Clinical records of 440 consecutive adult patients with newly diagnosed AML 15 hospitalized in our institution (the Hematology Division of the “Federico II” University of Naples) from 1 January 2002 to 31 December 2012 in order to receive cytotoxic agent induction courses for hematological remission 16, 17, 18, 19 were reviewed. All patients underwent central venous line insertion and antimicrobial prophylaxis with quinolones and azoles, as already reported 11.

During the study period, patients with severe neutropenia (absolute neutrophil count <500/*μ*l) and clinical signs suggestive of NEC, that is, fever (axillary temperature ≥38°C), diarrhea (>1 stool daily), and/or abdominal pain, were undergone ultrasonographic monitoring. Patients positive at US examination for pathologic bowel wall thickening (>4 mm in transversal scans for at least 30 mm length) were diagnosed with NEC, according to standardized criteria 1, 2, 8. Specific microbiological evaluations were also performed in these cases.

A retrospective study design was employed to collect clinical characteristics of diagnosed NEC episodes, but patients whose appropriate ultrasonographic and microbiological data were not available or receiving diagnosis of other diseases causing thickened intestinal wall were excluded from the analysis.

### Ultrasonographic and microbiological assessments

A hematologist (M.P., with more than 10 years of experience with US for intestinal assessment) carried out US scans of the four abdominal quadrants by using a scanner (iU22; Philips Healthcare, Bothell, WA) equipped with 1–5 MHz convex and 3–9 MHz linear probes. The ultrasonographic investigations were performed directly at the patient's bedside and were repeated every 3 days until complication resolution. The ileum and colon wall thickness was measured from outer wall to luminal surface, as previously reported 11, 12, 13, 14.

Blood cultures were performed every 24–48 h: the Vitek 2 automated system (bioMérieux, Marcy l'Etoile, France) was used for bloodstream isolate identification and antimicrobial susceptibility testing. Minimum inhibitory concentrations (MICs) were evaluated by using *E*‐test (BioMerieux) strips, and classified according to the European Committee on Antimicrobial Susceptibility Testing. Blood isolates of multidrug‐resistance (MDR) *Enterobacteriaceae* spp., such as extended‐spectrum *β*‐lactamase (ESBL)‐producing *Escherichia coli* and *Klebsiella pneumoniae* carbapenemase (KPC)‐producing *K. pneumoniae*, and vancomicin‐resistant *Enterococcus* spp. were confirmed using adjunct microbiological tests 20. Moreover, we monitored patients by performing multiple feces cultures and *Cytomegalovirus* tests.

### Antimicrobial management of infectious episodes

Therapeutic management of NEC episodes depended on the specific diagnostic phase. At febrile neutropenia onset, empirical approach consisted of monotherapy with a broad‐spectrum antibiotic or a combination of broad‐spectrum antibiotic plus anti‐gram‐negative drug followed by the addition of anti‐gram‐positive drug in selected cases on the basis of persistent fever and/or new clinical findings 1, 2. After diagnostic work‐up application, patients US positive for pathologic bowel wall thickening (i.e., imaging feature of inflamed/damaged bowel wall) were treated with at least two antibiotics displaying well‐documented gut penetration and/or activity against enteric bacteria (US‐driven approach) 21, 22, 23. In particular, the US parameters of bowel wall thickness ≥5 mm in transversal scans (for at least 30 mm length) triggered the use of a combination therapy, including *ß*‐lactam, aminoglycoside, glycopeptide, glycylcycline, and/or lipopeptide agents (at physician's discretion). In the case of positive microbiological findings, antiinfectious treatments were established according to the susceptibility of isolated pathogens (microbiology‐driven approach) 20, 21, 22, 23.

Anti‐gram‐negative, anti‐gram‐positive, and/or antifungal drugs were given at the recommended doses 21.

Bowel rest, total parenteral nutrition, albumin, packed red blood cells and/or platelets, and granulocyte colony‐stimulating factors (G‐CSF) were administered if clinically indicated 1, 2.

### Statistical analysis

The logistic regression model was used to compare survivor and nonsurvivor subgroups, thus to identify the predictors of outcome within 30 days of NEC onset (at the ultrasonographic appearance of pathologic intestinal mural thickening). The following variables were selected for the statistical analysis: age, gender, antileukemic protocols [containing standard‐dose (100 mg/m^2^ daily for at least 7 days), intermediate‐dose (200 mg/m^2^ daily for at least 7 days), and high‐dose (2000–6000 mg/m^2^ daily for at least 4 days) cytarabine] 16, 17, 18, 19, ultrasonographic features (bowel wall thickness >10 mm and >2 noncontiguous intestinal sites involved) 4, 8, microbiological findings (bloodstream infection and type of isolates from blood) 20, 21, 22, 23, antibacterial regimens with tigecycline during the empirical phase, US‐driven phase, and, in case of positive blood and/or stool cultures, microbiology‐driven phase (the impact of every combination antibiotic regimen was also evaluated in each phase), adjunct therapeutic modalities (systemic antifungal therapy, G‐CSF, bowel rest, and total parenteral nutrition), and severe neutropenia resolution (absolute neutrophil count >500/*μ*L).

Univariate analysis was first applied to assess the variables reported above. Multivariate analysis was used to identify independent risk factors for 30‐day mortality: in this analysis, variables found to be significant in univariate testing were incorporated with a stepwise approach. Continuous variables were compared with Student *t*‐test (for normally distributed variables) or the Mann–Whitney *U* test (for nonnormally distributed variables). Categorical variables were evaluated with the *χ*2 or two‐tailed Fisher exact test. Odds ratios (ORs) and 95% confidence intervals (CIs) were calculated for all associations that emerged. Results are expressed as median (range; continuous variables) or as percentages of the group from which they were derived (categorical variables).

Two‐tailed tests were used to determine statistical significance; a *P* < 0.05 was considered significant. Survival distribution function was estimated using the Kaplan–Meier product–limit method; nonparametric (log‐rank and Wilcoxon) tests were used to compare survival functions in different groups. All statistical analyses were performed with the SPSS program, version 18.0.

## Results

**Figure 1:**
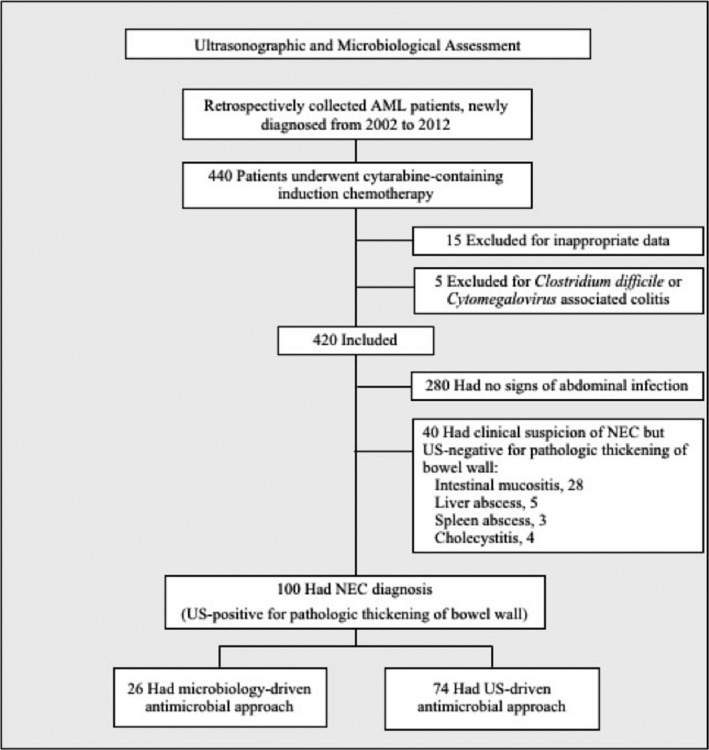
Study flowchart based on ultrasonography and microbiology evaluation. AML, acute myeloid leukemia; NEC, neutropenic enterocolitis; US, ultrasonography.

A total of 420 patients with AML who underwent induction chemotherapy courses for obtaining hematological remission from 2002 to 2012 were included in the study. Twenty other patients treated in this period were excluded owing to inappropriate data (*n* = 15) and *Clostridium difficile‐* or *Cytomegalovirus*‐associated colitis (*n* = 5; Fig. 1).

Patients baseline characteristics are detailed in Table 1: the median age was 50 years (range, 21–73 years), and 68.6% were male patients. The underlying hematological malignancy consisted of de novo (85%) or secondary (12%) AML, and blast crisis of chronic myeloprolipherative diseases (3%).

**Table 1:** Baseline characteristics of the entire patient population

Characteristic	No.
Number of patients	420
Gender	
Male/Female	288/132
Age, years	
Median (range)	50 (21–73)
Hematological malignancy	420
de novo AML	357
Secondary AML	50
Blast crisis of chronic myeloprolipherative diseases	13
Cytarabine‐based cytotoxic agent protocols	420
Standard‐dose cytarabine	
Induction course of EORTC‐GIMEMA AML‐10 trial	200
Intermediate‐dose cytarabine	
Induction course of HOVON‐SAKK trial	100
High‐dose cytarabine	
Induction course of FLAG‐Ida trial	71
Induction course of EORTC‐GIMEMA AML‐12 trial	49
Percentage of dose intensity administered	
Median (range)	90% (80–100%)

Unless otherwise specified data refer to the number of patients.

EORTC‐GIMEMA AML‐10 protocol: the schedule of induction course contained continuous *i.v*. infusion of cytarabine 100 mg/m^2^ daily for 10 days plus etoposide 100 mg/m^2^ per day by 1‐h *i.v*. infusion on days 1 through 5 plus daunorubicin 50 mg/m^2^ per day as a 5‐min *i.v*. infusion (*n* = 127 patients) or idarubicin 10 mg/m^2^ as a 5‐min *i.v*. infusion (*n* = 53 patients) or mitoxantrone 12 mg/m^2^ as a 30‐min *i.v*. infusion (*n* = 20 patients) on days 1, 3, and 5 [ref. 16].

HOVON‐SAKK protocol: the schedule of induction course contained continuous *i.v*. infusion of cytarabine 200 mg/m^2^ daily for 7 days plus idarubicin 12 mg/m^2^ daily as a 3‐h *i.v*. infusion on days 5 through 7 [ref. 17].

FLAG‐Ida protocol: the schedule of induction course contained cytarabine 2000 mg/m^2^ daily as 3‐h *i.v*. infusion for 4 days plus fludarabine 30 mg/m^2^ daily as 30 min *i.v*. infusion for 4 days plus idarubicin 12 mg/m^2^ daily as a 1‐h *i.v*. infusion on days 2 through 4 [ref. 18].

EORTC‐GIMEMA AML‐12 protocol: the schedule of induction course contained cytarabine 3000 mg/m^2^ every 12 h as 3‐h *i.v*. infusion on days 1, 3, 5, and 7 plus daunorubicin 50 mg/m^2^ per day as a 5‐minute *i.v*. infusion on days 1, 3, and 5 plus etoposide 50 mg/m^2^ per day by 1‐h *i.v*. infusion on days 1 through 5 [ref. 19].

AML, acute myeloid leukemia; EORTC, European Organization for Research and Treatment of Cancer; GIMEMA, Gruppo Italiano Malattie Ematologiche Maligne dell'Adulto; HOVON‐SAKK, Dutch‐Belgian Cooperative Group for Hemato‐Oncology and Swiss Group for clinical research; FLAG‐Ida, fludarabine–cytarabine–idarubicin plus granulocyte colony‐stimulating factors.

### Cytotoxic agent protocols

Patients underwent cytarabine‐based courses according to various protocols with antileukemic activity used in our Institution over a 10‐year period. In particular, 200 patients received schedules containing cytarabine at standard‐dose plus etoposide and daunorubicin (*n* = 127) or idarubicin (*n* = 53) or mitoxantrone (*n* = 20) according to the EORTC‐GIMEMA AML‐10 trial 16, 120 patients received schedules containing cytarabine at high‐dose plus fludarabine and idarubicin according to the FLAG‐Ida trial (*n* = 71) 18 or plus daunorubicin and etoposide according to the EORTC‐GIMEMA AML‐12 trial (*n* = 49) 19, and 100 patients received schedules containing cytarabine at intermediate‐dose plus idarubicin according to the HOVON‐SAKK trial 17. At least 80% dosages of the scheduled cytotoxic agents were administered in the patient population (Table 1).

### Classification of the episodes of neutropenia

During the aplastic phase following chemotherapy, all patients developed severe neutropenia with a median time of 10 days (range, 5–30 days). The clinical characteristics of neutropenic episodes are shown in Table 2. In particular, 187 patients (44.5%) had neutropenia without symptoms of infection, 93 patients (22.1%) developed microbiologically (*n* = 45) and/or clinically (*n* = 48) documented infections at respiratory tract (*n* = 53), central venous catheter (*n* = 31) or urinary tract (*n* = 9), and 140 patients (33.3%) had clinically and/or microbiologically documented intraabdominal infections.

**Table 2:** Clinical characteristics of postchemotherapy neutropenic episodes in the entire patient population

Characteristic	No.
Number of patients	420
Duration of severe neutropenia, days	
Median (range)	10 (5–30)
Neutropenic episodes without infectious symptoms	187
*Neutropenic episodes with extraabdominal infections*	93
Microbiological documented infections	45
Bacteremia	43
Gram positive	25
Coagulase‐negative *Staphylococcus*	20
*Enterococcus* spp.	5
Gram negative	18
*Eschericia Coli*	10
*Pseudomonas* spp.	4
*Klebsiella* spp.	4
Fungemia	2
*Candida albicans*	2
Clinically documented infections	48
Site/source of infection	
Upper respiratory tract	30
Lower respiratory tract	23
Central venous catheter	31
Urinary tract	9
*Neutropenic episodes with intraabdominal infections*	140
Microbiological documented infections	48
Bacteremia	45
Gram negative	33
*Escherichia Coli*	13
*Klebsiella* spp.	9
*Bacteroides* spp.	9
*Pseudomonas* spp.	2
Gram positive	12
*Enterococcus* spp.	8
*Streptococcus* spp.	2
*Staphylococcus* spp.	2
Fungemia	3
*Candida albicans*	2
*Candida tropicalis*	1
Site/source of infection	
Gut	128
Liver	5
Gall bladder	4
Spleen	3
Abdominal symptoms (plus fever)	140
Diarrhea	36
Intestinal bleeding	29
Abdominal pain	25
Acute abdomen^a^	20
Abdominal pain + diarrhea	15
Septic shock^a^	15

Unless otherwise specified data refer to the number of patients.

a According to the diagnostic criteria reported in ref. 22, 23.

US of abdomen and pelvis was performed within 24 h of complication onset in all the patients with clinical suspicion of NEC (*n* = 140). Among these, 40 patients were found to have US negative for pathologic thickening of bowel wall (thickness ≤4 mm in transversal scans, 34 cases; or thickness >4 mm in transversal scans for <30 mm length, 6 cases) and thus excluded from NEC diagnosis (intestinal mucositis, *n* = 28; hepatic and/or splenic abscesses, *n* = 8; cholecystitis, *n* = 4). The remaining 100 patients were found US positive for pathologic thickening of bowel wall and were diagnosed with NEC according to predefined criteria (Fig. 1). A mean number of two US examinations was performed per episode of intraabdominal infection to make NEC diagnosis (range, 1–4).

### Epidemiological and ultrasonographic aspects of NEC

The incidence rate of NEC was 23.8% (100 of 420 patients). Chemotherapies protocols containing high‐dose cytarabine, standard‐dose cytarabine, and intermediate‐dose cytarabine preceded NEC onset in 51 patients, 30 patients, and 19 patients, respectively.

**Figure 2:**
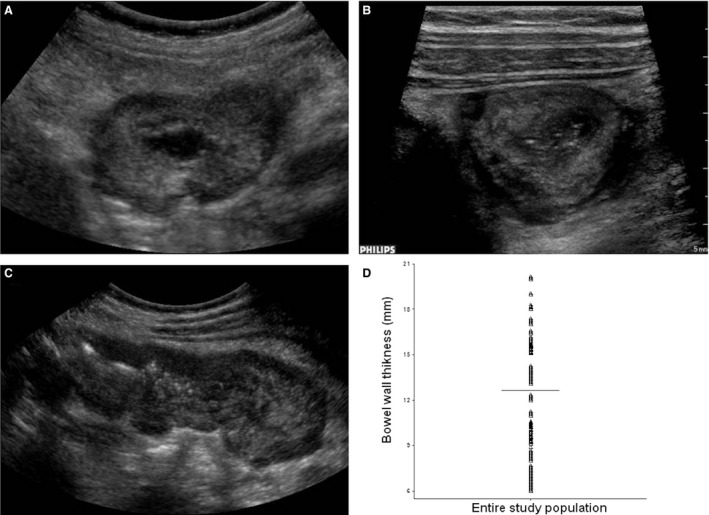
A‐B‐C‐D. Ultrasonographic features of neutropenic enterocolitis. (A) Transverse scan with 5‐1‐MHz convex probe showing a rounded mass due to severe bowel wall thickening (16 mm) of the cecum. (B) Transverse scan with 9‐3‐MHz linear probe identifying different wall layers, in particular hypoechoic central portion (virtual lumen and mucosa), wide hyperechoic submucosal, and hypoechoic periphery (muscularis mucosa) in the same case. (C) Longitudinal scan with 5‐1‐MHz convex probe showing >4 mm thickness for at least 30 mm in length, in the same case. (D) Median bowel wall thickness (12 mm; range, 6–20 mm) of the entire patient population with neutropenic enterocolitis.

At US scanning, pathologic bowel wall thickening was found to be localized in the small bowel (30 patients), the large bowel (28 patients), and both (42 patients). The median wall thickness was 12 mm with a range of 6–20 mm, for a median length in longitudinal scans of 4 cm (range, 3–5 cm); mural thickening appeared in different patterns (Fig. 2). No intramural or free abdominal air was noted, whereas free abdominal fluid was observed in 60 patients.

### Microbiological surveillance of NEC

Overall, only for 26 of the 100 patients with NEC blood and/or stool cultures yielded pathogens (microbiology‐documented NEC). Gram‐negative rods were found in 18/26 (69%) patients. *Escherichia coli* was present in 10 patients; whereby, there were 5 cases of ESBL‐producing strains only susceptible to tigecycline (MICs ≤1 mg/L), meropenem (MICs ≤ 1 mg/L), amikacin (MICs ≤8 mg/L), and/or colistin (MICs ≤ 0.5 mg/L). *Klebsiella pneumoniae* was present in 7 patients; among them, there were 3 cases of KPC‐producing strains only susceptible to tigecycline (MICs ≤ 1 mg/L) and/or colistin (MICs ≤ 2 mg/L). *Pseudomonas aeruginosa* was present in one case, only susceptible to colistin (MIC 0.5 mg/L). Gram‐positive bacteria were cultured in 6/26 (23%) patients: *Streptococcus mutans* in three patients, and vancomicin‐, penicillin‐, and gentamicin‐resistant *Enterococcus faecium* in 3 cases. All Gram‐positive isolates were susceptible to tigecycline (MICs ≤ 0.5 mg/L) and/or daptomycin (MICs ≤ 0.5 mg/L). In 2/26 (8%) patients, the organism responsible for bloodstream infection was *Candida albicans,* susceptible to caspofungin and/or amphotericin B.

For the remaining 74 patients with NEC, the microbiological surveillance did not provide isolates.

### Antimicrobial treatment of NEC

At neutropenic fever onset, antibacterial treatments were empirically given to all patients according to febrile neutropenia‐driven approach (monotherapy with a *ß*‐lactam agent, *n* = 4; or two‐antibiotic combinations including a *ß*‐lactam agent, *n* = 96). Only three patients were being treated in this phase with combination regimens including tigecycline.

After ultrasonographic NEC diagnosis, reported 24–264 h (median 84 h) from neutropenic fever onset, antibacterial regimens of NEC were given to 74 patients in the absence of microbiological isolates using drugs displaying gut penetration and/or broad activity against enteric bacteria (US‐driven approach), and consisted of three‐antibiotic combinations for 57 patients (ceftazidime plus amikacin and teicoplanin, 18 cases; piperacillin–tazobactam plus amikacin and teicoplanin, 14 cases; meropenem plus amikacin and teicoplanin, 14 cases; and tigecycline plus meropenem and daptomycin, 11 cases), and two‐antibiotic combinations for the remaining 17 patients (piperacillin–tazobactam plus amikacin, 7 cases; tigecycline plus meropenem, 7 cases; and tigecycline plus piperacillin–tazobactam, 3 cases).

In the remaining 26 patients with NEC and bacterial and/or fungal isolates, the definitive antimicrobial regimens were given according to antibiograms (reported 96–240 h, median 144 h, after neutropenic fever onset) and consisted of three‐antibiotic combinations for 14 patients (meropenem plus amikacin and colistin, 12 cases; tigecycline plus meropenem and daptomycin, 2 cases), two‐antibiotic combinations for 10 patients (tigecycline plus meropenem, 6 cases; and meropenem plus daptomycin, 4 cases), and liposomal amphotericin B for the 2 patients with *Candida* from blood.

Adjunct therapeutic modalities included bowel rest (90 patients), intravenous infusions of antifungal drugs [given empirically to 38 patients after 6 median days (range, 4–8 days) from neutropenic fever onset (liposomal amphotericin B, 18 patients; azoles, 10 patients; and caspofungin, 10 patients)], G‐CSF (36 patients), and total parenteral nutrition (32 patients).

Antimicrobial dosages were adjusted on the basis of renal, liver, and/or neurological toxicity, if necessary.

The overall median duration of antimicrobial therapy was 16 days (range, 2–30 days).

### Mortality rate of NEC and predictors of outcome

Overall, 30 days after NEC onset, 23 patients died from acute abdomen (10 cases), massive intestinal bleeding (8 cases), and/or septic shock (5 cases). Thus, NEC attributable mortality rate was 23%.

**Figure 3:**
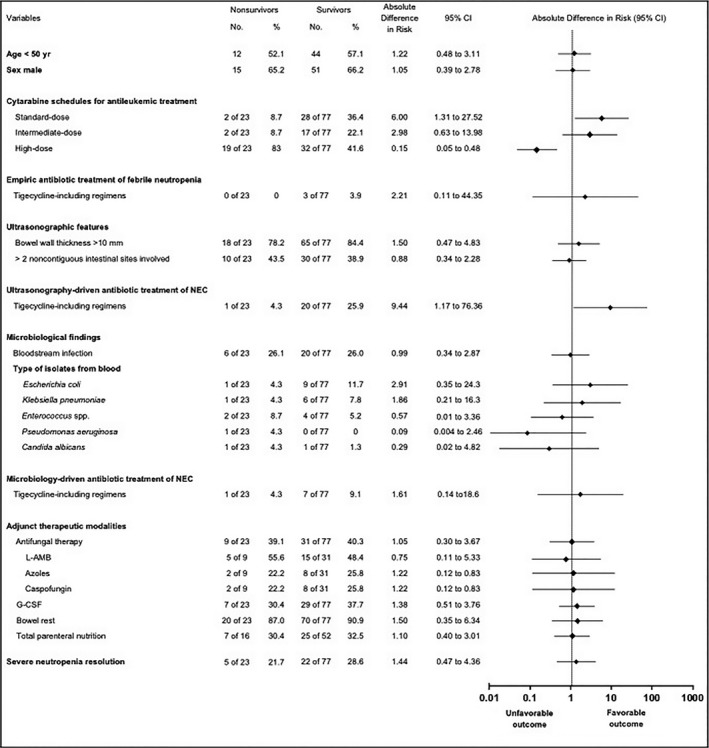
Subgroup analyses of overall survival for several variables associated with death among the entire patient population with neutropenic enterocolitis. Rates and absolute differences in the risk of mortality are given. L‐AMB, liposomal amphotericin B; G‐CSF, granulocyte colony‐stimulating factors; Ultrasonography‐driven antibiotic treatment of NEC = antibacterial regimens, promptly given at ultrasonographic appearance of pathologic intestinal mural thickening. Azoles =  fluconazole in 6 cases and voriconazole in 4 cases. *Enterococcus* spp. included 3 cases of *Streptococcus mutans* infections and 3 cases of *Enterococcus faecium* infections.

Univariate analysis revealed significant differences between the nonsurvivor and survivor subgroups (Fig. 3). Patients in the nonsurvivor subgroup were more likely to have received induction chemotherapy courses based on high‐dose cytarabine (*P *<* *0.001). Survivors were more likely to have received induction chemotherapy courses based on standard‐dose cytarabine (*P *=* *0.011), and US‐driven NEC treatment with antibiotic regimens including tigecycline (*P *=* *0.025). Logistic regression analysis identified induction chemotherapy courses based on high‐dose cytarabine (OR: 0.109; 95% CI: 0.032–0.364; *P *<* *0.001) as the independent predictor of 30‐day mortality, whereas US‐driven NEC treatment with antibiotic combinations including tigecycline (OR: 13.161; 95% CI: 1.587–109.17; *P *=* *0.017) was associated with the lowest risk of mortality.

At univariate statistical analysis, comparing the height of every single antibiotic drug, used or not in combination with tigecycline, none has been shown to have a protective effect in reducing NEC‐related mortality (data not shown). Moreover, no significant difference was observed between the survivor and nonsurvivor subgroups with regard to age, gender, degree of bowel wall thickness, and number of intestinal sites involved as detected by ultrasonographic assessment, microbiological findings, in particular bloodstream infections due to ESBL‐producing *Escherichia coli* (survivor subgroup: 4/77 patients, 5.2%; nonsurvivor subgroup: 1/23 patients, 4.3%; *P *=* *0.87), KPC‐producing *Klebsiella pneumoniae* (survivor subgroup: 2/77 patients, 2.6%; nonsurvivor subgroup: 1/23 patients, 4.3%; *P *=* *0.66), and MDR *Enterococcus faecium* (survivor subgroup: 2/77 patients, 2.5%; nonsurvivor subgroup: 1/23 patients, 4.3%; *P *=* *0.66), adjunct therapeutic modalities (such as antifungal therapy), and severe neutropenia resolution.

## Discussion

To the best of our knowledge, we herein reported the largest series of patients suffering from NEC following chemotherapy treatment for AML 1, 2. Four main findings of our study require further attention.

**Figure 4:**
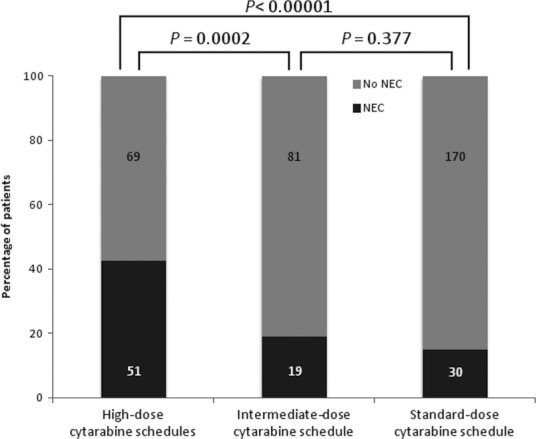
Incidence of NEC in the subgroups of patients according to the schedules of cytarabine included in the induction protocols for hematological remission. The numbers of patients affected and not affected are inside the columns.

First, antileukemic protocols containing high‐dose cytarabine were the key factor for the development of NEC and its related mortality rate. Cytarabine is a powerful cytotoxic agent available for the remission of AML in combination with anthracyclines, and/or etoposide and/or fludarabine 24. The pharmacodynamic properties of cytarabine led to its use with different dosages and modalities according to the schedules included in the frame of antileukemic induction program over a 10‐year period: standard‐dose, intermediate‐dose, or high‐dose with variable time of i.v. infusions 16, 17, 18, 19. We analyzed the impact on intestinal toxicity of the various schedules and obtained significant data. In fact, 42% of patients treated with high‐dose cytarabine 18, 19 suffered from NEC, versus 19% and 15% of patients treated with intermediate‐dose and standard‐dose cytarabine 16, 17, respectively (*P *<* *0.001; Fig. 4). Most importantly, robust dosages of cytarabine resulted in about fourfold increase in mortality rate compared with the other schedules (37.2% with high‐dose vs. 10.5% or 6.6% with intermediate‐ or standard‐dose, respectively; *P ≤ *0.03) and <70% survival rate at 30 days after NEC onset (Fig. 3). Noteworthy, all cases of acute abdomen occurred in the subset of patients who had received antileukemic protocols containing high‐dose cytarabine. This severity of NEC suggests that the robust dosages of cytarabine are involved in a direct damage on bowel wall.

Second, gram‐negative and gram‐positive pathogens with MDR emerged among the 26 patients with microbiology‐documented NEC. In fact, 17 cases carried *Enterobacteriaceae* spp. and 6 cases carried gram‐positive cocci: 47% of the strains of *Enterobacteriaceae* spp. produced ESBL or carbapenemase, and 50% of the strains of gram‐positive cocci showed glycopeptide resistance 20. These findings highlight the pathogenic role of difficult‐to‐treat emerging enteric bacteria for the development of NEC, and the pressing need of new antibacterial drugs displaying specific activity against these species 1, 9, 10, 25.

Third, systemic use of modern US as part of the diagnostic work‐up of patients with neutropenic fever and cancer, especially patients treated with schedules containing high dosages of cytarabine 19, was particularly valuable to make diagnosis of NEC, identifying bowel wall thickening, the true warning sign of NEC at an early stage when conservative treatment would be maximally effective. In our series, a bowel wall thickness ≥5 mm (for at least 30 mm length) was the pragmatic criterion to start promptly vigorous medical treatment consisting of antibiotic regimens including at least two drugs with well‐documented gut penetration and/or activity against emerging enteric pathogens responsible for complicated intraabdominal infections 11, 12, 13, 14. US‐driven approach allowed to administer adequate antimicrobial therapy after a median of 3.5 days from the appearance of fever versus a median of 6 days (from the appearance of fever) by using microbiology‐driven approach (*P *=* *0.03, by *t*‐test).

**Figure 5:**
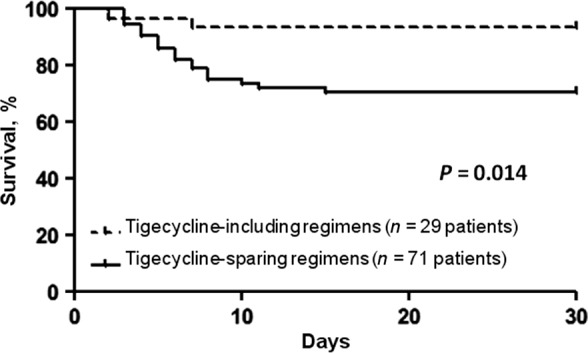
Kaplan–Meier curves. The impact of tigecycline‐including combination regimens (dotted line) versus tigecycline‐sparing combination regimens (solid line) on 30‐day mortality of patients with neutropenic enterocolitis. Days = days from neutropenic enterocolitis onset.

Fourth, the most adequate antimicrobial therapy consisted of a combination regimen with tigecycline. This kind of schedule, significantly reduced NEC‐related morbidity and mortality. In fact, the multivariate analysis demonstrated that antibiotic combination therapy including tigecycline was the only factor significantly linked to a reduced risk of death. The 30‐day survival rate associated with regimens containing this particular drug was 93% (27 of 29) vs. 70% (50 of 71) in patients who were treated with regimens sparing tigecycline (*P *=* *0.014; Fig. 5). This finding is expected due to the peculiar characteristics of tigecycline. In the study by Rubino et al., tigecycline showed excellent penetration into the ileum and colon wall and peritoneal fluid: the drug exposure at bowel tissue was significantly greater than that in serum, likely providing an adequate AUC (Area Under the Curve)‐to‐MIC ratio for a good bactericidal activity against enteric bacterial strains 26. In our study, as part of a two‐ or three‐antibiotic combination therapy along with tigecycline, a *β*‐lactam agent with antipseudomonal coverage was always used. Meropenem was the drug most frequently combined with tigecycline (26 cases; among them, one died), then daptomycin (13 cases; among them, none died), and piperacillin–tazobactam (3 cases; among them, one died). There exist in vitro data and case report studies on the synergistic activity of tigecycline with *β*‐lactam and/or other classes of antibacterial agents, for example, meropenem and/or daptomycin 27, 28. Characteristics of patients receiving tigecycline‐including regimens versus those receiving tigecycline‐sparing regimens in our study are detailed in Table 3.

**Table 3:** Characteristics of patients treated with antibiotic regimens including or sparing tigecycline

	Tigecycline‐including regimens	Tigecycline‐sparing regimens
Nr. of patients	29	71
*Acute myeloid leukemia‐related factors*		
Baseline risk classification		
Karyotype cytogenetics		
Favorable	6 (20.7)	16 (22.5)
Intermediate	15 (51.7)	34 (47.9)
Unfavorable	8 (27.6)	21 (29.6)
Secondary acute myeloid leukemia	1 (3.4)	3 (4.2)
Blast crisis	—	1 (1.4)
Failure to achieve CR by day 15 and/or by day 28	7 (24.1)	16 (22.5)
Host‐related factors at start of remission induction chemotherapy		
ECOG performance status ≥ 2	—	1 (1.4)
Patient age ≥ 50	12 (41.4)	32 (45.1)
Gender (Male)	18 (62)	48 (67.6)
Subjects with inadequate renal function^a^	—	1 (1.4)
Subjects with inadequate liver function^b^	1 (3.4)	2 (2.8)
Subjects with clinically significant cardiac disease^c^	—	1 (1.4)
Year of NEC diagnosis		
Median (range)	2010 (2007–2012)	2005 (2002–2012)
Ultrasonography features		
Bowel wall thickness		
Median (range), mm	12.5 (6–20)	11 (6–19)
Microbiological findings	8 (27.6)	18 (25.3)
*Escherichia coli*	3 (10.3)	7 (9.8)
ESBL‐producing strains	3 (10.3)	2 (2.8)
*Klebsiella pneumoniae*	3 (10.3)	4 (5.6)
KPC‐producing strains	3 (10.3)	—
*Streptococcus mutans*	—	3 (4.2)
*Enterococcus faecium*	2 (6.9)	1 (1.4)
Glycopeptide‐resistant	2 (6.9)	1 (1.4)
*Pseudomonas aeruginosa*	—	1 (1.4)
*Candida albicans*	—	2 (2.8)
Abdominal surgery	—	6 (8.4)
Admission in intensive care unit	1 (3.4)	7 (9.8)
Death	2 (6.9)	21 (29.6)

Unless otherwise specified data refer to the number of patients.

CR, complete remission; ECOG, Eastern Cooperative Oncology group.

All patients received antimicrobial prophylaxis with oral levofloxacin (500 mg daily) and antimold agents (oral itraconazole (400 mg daily) or posaconazole [600 mg daily]).

a Serum creatinine ≥ 1.5 mg/dL or eGFR < 59 mL/min.

b Aspartate Aminotranferase (AST) and/or Alanine Aminotransferase (ALT) and/or direct bilirubin ≥ 2.5 ×  upper limit of normal (ULN).

c Left ventricular ejection fraction ≤ 45%.

Our study has limitations, however. First, it is a single‐center retrospective study which limits the value of the results, in particular, inter‐observer and interequipment US reproducibility should be tested. Therefore, our findings need to be validated in a prospective multicenter trial. Second, characteristics of involved infectious pathogens depend on local epidemiology, local susceptibility, or resistance patterns, and previous antimicrobial exposure. Recent data showing the spread of gram‐negative and gram‐positive enteric strains with increased resistance rates for tigecycline have been reported 29. Furthermore, although the low frequency of *Pseudomonas aeruginosa* infections in our setting, antipseudomonal coverage is very important in high‐risk patients with neutropenic fever 10, 21. Third, the study spans 2002–2012, but tigecycline was not commercially available until 2007. Thus, the increased survival may be due to a general improvement in care for recent patients receiving tigecycline. Fourth, traditional microbiological tests, employed in the study, led to positive cultures late (median: 6 days after neutropenic fever onset). The use of new microbiology tools (such as, septifast test, and/or MALDI‐TOF mass spectrometry) 30 could improve the antimicrobial strategy for NEC. Finally, recovery from neutropenia did not effect outcome in our series. Larger studies are needed to confirm this observation.

In conclusion, under optimal study conditions and with an experienced operator, modern US is rapid, safe, effective, and low cost for abdominal–pelvis exploration 17, 18, 19, in particular for scanning bowel wall of patients who have received cytarabine‐containing chemotherapy protocols for the remission of underlying hematological malignancy 12, 13, 14, 15. At NEC diagnosis, prompt administration of vigorous antibiotic regimens with drug combination which have broad antimicrobial coverage and gut penetration, specifically those also including tigecycline, may be very effective.

## Conflict of Interest

The authors declare that they have no conflict of interest.
